# Special Issue “Molecular Targets in Gastrointestinal Diseases”

**DOI:** 10.3390/ijms27198463

**Published:** 2026-09-23

**Authors:** Changwon Yang

**Affiliations:** Department of Science Education, Ewha Womans University, Seoul 03760, Republic of Korea; cyang@ewha.ac.kr; Tel.: +82-2-3277-2688

Gastrointestinal (GI) diseases encompass a broad spectrum of disorders, ranging from chronic inflammatory conditions to malignancies, and are characterized by considerable molecular and cellular heterogeneity. Despite substantial advances in their diagnosis and treatment, many GI diseases remain difficult to manage. Their development and progression involve complex interactions among genetic and epigenetic alterations, dysregulated signaling pathways, immune responses, and the intestinal microbiota. A deeper understanding of these mechanisms is therefore essential for clarifying disease pathogenesis. It is equally important for identifying actionable targets and biomarkers that may improve therapeutic efficacy, overcome treatment resistance, and enable more precise patient stratification. In this context, the Special Issue “Molecular Targets in Gastrointestinal Diseases” brings together two original research articles and three reviews that address GI cancers and inflammatory bowel disease (IBD). These contributions approach molecular targeting from three complementary angles: cancer-associated signaling and therapeutic resistance, microbiome-related alterations, and immune regulatory mechanisms.

A major theme of this Special Issue is the identification of tumor-intrinsic molecular vulnerabilities that may be exploited therapeutically in GI cancers. Chen et al. reviewed the NIMA-related kinase (NEK) family and summarized the dysregulated expression and functional involvement of its members in tumor progression and drug resistance across GI malignancies. NEK2 emerged as the most extensively studied and particularly promising candidate. It is consistently overexpressed across GI cancer types, and a NEK2 inhibitor, T-1101 tosylate, has entered phase I clinical trials (Contribution 1). The review also noted that NEK2 phosphorylates programmed death-ligand 1 (PD-L1) and confers resistance to immunotherapy in pancreatic cancer, which links this kinase to tumor immune evasion [1]. Complementing this target-identification perspective, Park et al. investigated a strategy to enhance the efficacy of 5-fluorouracil (5-FU) in gastric cancer by targeting a mechanism of chemoresistance. Treatment with 5-FU alone induced a compensatory increase in thymidylate synthase (TS), a known resistance mechanism. The histone deacetylase (HDAC) inhibitor panobinostat suppressed basal TS expression and abolished this 5-FU-induced upregulation, and the combination produced synergistic cytotoxicity in Chou–Talalay analysis (Contribution 2). Panobinostat also downregulated c-Myc and a network of cell cycle regulators, indicating that its chemosensitizing effect extends beyond a single target. These two studies represent complementary routes to molecularly guided cancer therapy. One route identifies potentially actionable oncogenic kinases. The other modulates the pathways that sustain resistance to established chemotherapy. Viewed together, they indicate that the therapeutic relevance of a molecular target depends on its contribution to tumor growth and, just as importantly, on its potential to improve the response to existing treatment.

Beyond tumor-intrinsic signaling, the gut microbiome has become an important component of the molecular landscape of GI diseases. Kondo et al. compared mucosa-associated and luminal microbiota in pediatric ulcerative colitis (UC) using colonic lavage fluids collected from four colonic sites during endoscopy, a less invasive alternative to multiple biopsies in children. In non-IBD controls, the mucosa-associated communities differed clearly from those in feces, whereas this distinction was lost in UC. The authors interpreted this finding as evidence of a compromised mucus layer that no longer separates mucosal and luminal bacteria. Regional heterogeneity of the mucosa-associated microbiota along the colon increased with disease activity and mucosal inflammation, and butyrate-producing genera such as *Faecalibacterium* and *Blautia* were depleted in UC (Contribution 3). These observations show that the sampling site and the tissue context shape how microbiome alterations should be interpreted in intestinal disease. Extending this concept to GI cancer, Kim et al. reviewed microbiome signatures in advanced gastric cancer and their potential roles in risk stratification, therapeutic guidance, and prognosis. Specific taxa were connected to defined mechanisms. *Fusobacterium nucleatum* activates IL-17/NF-κB signaling and recruits PD-L1-expressing neutrophils that dampen cytotoxic T cell activity. In contrast, *Bifidobacterium* and *Lactobacillus rhamnosus* GG enhance dendritic cell and cytotoxic T cell function through cGAS-STING-dependent type I interferon signaling and may improve responses to immune checkpoint inhibitors (Contribution 4) [2,3]. The review also identified substantial barriers to clinical translation, including methodological heterogeneity, limited evidence for causality, and the scarcity of prospective clinical studies. These two contributions present the microbiome as a context-dependent ecosystem closely linked to mucosal integrity and host immunity, and not simply as a collection of disease-associated biomarkers. Clinically useful microbiome-based targets will therefore require careful choice of sampling strategy, methodological standardization, and validation in well-characterized patient populations.

Immune regulation adds another layer of molecular control, particularly in chronic inflammatory conditions such as IBD. Sznurkowska reviewed the role of regulatory T cells (Tregs), which are widely regarded as key mediators of immune tolerance and suppression of excessive inflammation. The available evidence in human IBD, however, does not consistently demonstrate quantitative deficits or intrinsic functional defects of Tregs (Contribution 5). Inflamed mucosa is, in fact, enriched in Tregs, and the studies that examined treatment-naïve pediatric patients reported increased circulating and intestinal Tregs at disease onset. Clinical evidence for Treg-based therapy is also limited, as only one clinical trial of Treg infusion in Crohn’s disease has been completed, and its results did not provide sufficient evidence to establish efficacy or safety [4]. The persistence of inflammation despite abundant Tregs raises questions about effector cell resistance to suppression, the antigen specificity of the Treg clones present in the mucosa, and Treg plasticity under inflammatory conditions. This gap between strong biological plausibility and uncertain clinical relevance reflects a broader challenge in molecular target development. Molecules or cell populations implicated in disease pathogenesis do not necessarily translate into effective therapeutic targets. In IBD, immune dysregulation most likely arises from interactions among regulatory and effector immune cells, mucosal barrier integrity, and microbial stimuli, and not from dysfunction of a single immune component. The distinct Treg profile of treatment-naïve children also resonates with the pediatric UC study in this issue, and both point to disease stage and patient age as variables that must be considered in target validation. Future efforts to exploit immune regulatory pathways will therefore require precise functional characterization, disease-context-specific validation, and evidence linking target modulation to clinically meaningful outcomes.

Taken together, the contributions to this Special Issue show that molecular targeting in GI diseases extends well beyond the identification of a single dysregulated molecule. Actionable targets may reside in tumor-intrinsic signaling networks or in adaptive mechanisms that limit the effectiveness of established therapies (Contribution 1) (Contribution 2). In inflammatory and neoplastic disorders alike, the disease context is further shaped by the microbiome and the host immune system (Contribution 3) (Contribution 4) (Contribution 5). The contributions also reveal a mechanistic thread that runs across these themes. Butyrate-producing taxa were depleted in the mucosa-associated microbiota of pediatric UC (Contribution 3). In gastric cancer models, short-chain fatty acids such as butyrate lower PD-L1 and IL-10 expression in tumor-associated macrophages, support Tregs, and strengthen epithelial integrity (Contribution 4) [5,6]. Microbial metabolites therefore provide a plausible link between the microbiome and mucosal immunity that neither field can fully address in isolation. The clinical relevance of a molecular target thus depends on the biological context in which it operates, including tumor genotype and phenotype, treatment exposure, microbial ecology, and immune regulation. Future advances will depend on discovering new targets and, equally, on defining the contexts in which those targets are functionally relevant and therapeutically actionable.

Several research priorities emerge from these contributions, and they share a common challenge. Promising molecular associations and mechanistic findings must be translated into robust biomarkers and therapeutic strategies through rigorous functional and clinical validation. In GI cancers, NEK2 inhibitors such as T-1101 tosylate should be evaluated in clinically relevant GI cancer models. The link between NEK2 and PD-L1 further suggests that NEK2 inhibition should be tested in combination with immune checkpoint blockade (Contribution 1). The chemosensitizing effect of panobinostat requires confirmation in 5-FU-resistant cells and in vivo, and TS expression deserves evaluation as a biomarker for selecting patients likely to benefit from HDAC inhibitor combinations (Contribution 2). For the microbiome, mucosa-associated and luminal communities should be sampled and reported separately. Less-invasive approaches such as colonic lavage need validation against biopsy-based methods in larger and longitudinal cohorts that include healthy controls (Contribution 3). Shotgun metagenomics, standardized analytical pipelines, and experimental models capable of testing causality will be needed to separate causal drivers from disease-associated changes (Contribution 4). Prospective trials should then test whether microbiome-informed interventions improve responses to immunotherapy. In immune regulation, studies of treatment-naïve patients at disease onset are needed, together with better definition of Treg subsets and plasticity in inflamed mucosa. Evaluation of effector cell resistance to suppression will also help to clarify whether Tregs represent a viable therapeutic target (Contribution 5). Finally, microbial metabolites merit direct investigation as a shared mechanism connecting the microbiome, mucosal immunity, and treatment response.

Progress in this field will ultimately depend on integrating mechanistic discovery with appropriate disease models, patient stratification, and prospective clinical investigation. This Special Issue brings together complementary perspectives on cancer signaling, treatment resistance, the microbiome, and immune regulation. In doing so, it offers a framework for moving molecular insights toward more precise and effective approaches to the diagnosis and treatment of GI diseases.

## Abbreviations

The following abbreviations are used in this manuscript:

GI	Gastrointestinal
NEK	NIMA-related kinase
PD-L1	Programmed death-ligand 1
5-FU	5-fluorouracil
TS	Thymidylate synthase
HDAC	Histone deacetylase
UC	Ulcerative colitis
IBD	Inflammatory bowel disease
Tregs	Regulatory T cells
